# Risk-adjusted therapy for pediatric non-T cell ALL improves outcomes for standard risk patients: results of JACLS ALL-02

**DOI:** 10.1038/s41408-020-0287-4

**Published:** 2020-02-27

**Authors:** Daiichiro Hasegawa, Toshihiko Imamura, Keiko Yumura-Yagi, Yoshihiro Takahashi, Ikuya Usami, So-ichi Suenobu, Shinichiro Nishimura, Nobuhiro Suzuki, Yoshiko Hashii, Takao Deguchi, Akiko Moriya-Saito, Koji Kato, Yoshiyuki Kosaka, Masahiro Hirayama, Akihiro Iguchi, Hirohide Kawasaki, Hiroki Hori, Atsushi Sato, Tooru Kudoh, Tatsutoshi Nakahata, Megumi Oda, Junichi Hara, Keizo Horibe, for the Japan Association of Childhood Leukemia Study Group (JACLS)

**Affiliations:** 1 Department of Hematology/Oncology, Hyogo Prefectural Children’s Hospital, Kobe, Japan; 2 Department of Pediatrics, Kyoto Prefectural University of Medicine, Graduate School of Medical Science, Kyoto, Japan; 3 Clinical Research Center, National Hospital Organization Nagoya Medical Center, Nagoya, Japan; 4 Yumura Clinic, Osaka, Japan; 5 Department of Pediatrics, Aomori Prefectural Central Hospital, Aomori, Japan; 6 Department of Pediatric Hematology and Oncology, Hyogo Prefectural Amagasaki General Medical Center, Hyogo, Japan; 7 Division of General Pediatrics and Emergency Medicine, Department of Pediatrics, Oita University, Oita, Japan; 8 Department of Pediatrics, Hiroshima University Hospital, Hiroshima, Japan; 9 Department of Pediatrics, Hokkaido Medical Center for Child Health and Rehabilitation, Sapporo, Japan; 10 Department of Pediatrics, Osaka University, Suita, Japan; 11 Department of Pediatrics, Mie University, Tsu, Japan; 12 Department of Hematology Oncology, Children’s Medical Center, Japanese Red Cross Nagoya First Hospital, Nagoya, Japan; 13 Department of Pediatrics, Hokkaido University Hospital, Sapporo, Japan; 14 Department of Pediatrics Kansai Medical University, Hirakata, Japan; 15 Department of Hematology/Oncology, Miyagi Children’s Hospital, Sendai, Japan; 16 Saiseikai Nishiotaru Hospital, Otaru, Japan; 17 Department of Clinical Application, Center for iPS Cell Research and Application (CiRA), Kyoto University, Kyoto, Japan; 18 Department of Pediatrics, Okayama University, Okayama, Japan; 19 Department of Pediatric Hematology/Oncology, Osaka City General Hospital, Osaka, Japan

**Keywords:** Acute lymphocytic leukaemia, Randomized controlled trials

## Abstract

This study was a second multicenter trial on childhood ALL by the Japan Childhood Leukemia Study Group (JACLS) to improve outcomes in non-T ALL. Between April 2002 and March 2008, 1138 children with non-T ALL were enrolled in the JACLS ALL-02 trial. Patients were stratified into three groups using age, white blood cell count, unfavorable genetic abnormalities, and treatment response: standard risk (SR), high risk (HR), and extremely high risk (ER). Prophylactic cranial radiation therapy (PCRT) was abolished except for CNS leukemia. Four-year event-free survival (4yr-EFS) and 4-year overall survival (4yr-OS) rates for all patients were 85.4% ± 1.1% and 91.2% ± 0.9%, respectively. Risk-adjusted therapy resulted in 4yr-EFS rates of 90.4% ± 1.4% for SR, 84.9% ± 1.6% for HR, and 66.5% ± 4.0% for ER. Based on NCI risk classification, 4yr-EFS rates were 88.2% in NCI-SR and 76.4% in NCI-HR patients, respectively. Compared to previous trial ALL-97, 4yr-EFS of NCI-SR patients was significantly improved (88.2% vs 81.2%, log rank *p* = 0.0004). The 4-year cumulative incidence of isolated (0.9%) and total (1.5%) CNS relapse were significantly lower than those reported previously. In conclusion, improved EFS in NCI-SR patients and abolish of PCRT was achieved in ALL-02.

## Introduction

Acute lymphoblastic leukemia (ALL) is the most common malignancy in childhood, accounting for approximately 25% of pediatric cancers and almost 75% of pediatric leukemias. Treatment results in pediatric ALL are one of the true successes of modern clinical oncology, with 5-year survival rates of almost 90% achieved by the application of intensive multiagent chemotherapeutic regimens and risk-adjusted treatments^1–3^. From 1997 to 2002, the Japan Association of Childhood Leukemia Study Group (JACLS) conducted the first clinical trial, the JACLS ALL-97 trial, resulting in 4-year event-free survival (4yr-EFS) rates of 81.3% for standard risk (NCI-SR) and 71% for high risk (NCI-HR) patients^4^. Comparison of these results with those reported from major western study groups indicated that the EFS of NCI-SR patients was unsatisfactory^5,6^. Thus, to improve EFS of NCI-SR patients, refined risk assignment through the analysis of prednisolone responses, which were established by the Berlin–Frankfurt–Munster (BFM) group^6^, were employed in the JACLS ALL-02 study.

It is also important to reduce late treatment-related effects, which can occur in more than two-thirds of long-term survivors, particularly patients treated with prophylactic cranial irradiation therapy (pCRT). In this context, the criteria for use of pCRT should be reconsidered, as one-third of patients participating in the JACLS ALL-97 study received pCRT as central nervous system (CNS)-directed therapy^4^. Thus, we attempted to abandon pCRT for non-T ALL by intensifying systemic chemotherapy and implementing protracted triple intrathecal chemotherapy in the JACLS ALL-02 trial. Herein, we report the outcomes of patients with non-T ALL treated in the JACLS ALL-02 trial.

## Methods

### Patients

Between 1 April 2002 and 31 March 2008, 1252 consecutive patients aged 1–18 years with newly diagnosed ALL were enrolled in JACLS ALL-02. Patients with Ph+ ALL, mature B-ALL, and NK-leukemia were excluded because they underwent other specific protocols. Patients were treated in 102 participating study centers in Japan. Informed consent was obtained from the guardians of each patient, in accordance with the Declaration of Helsinki. The trial was approved by the Ethical Committees of all participating institutions. The median follow-up period for the patients analyzed were 6.6 years. Follow-up data were fixed for analysis in March 2015.

### Diagnosis

A diagnosis of ALL was established if ≥25% lymphoblasts were present in bone marrow (BM). BM and peripheral blood (PB) smears, as well as cerebrospinal fluid (CSF) cytospin preparations, were reviewed in each study center. CNS involvement was diagnosed as CSF pleocytosis of >5 cells/μL and the presence of recognizable blast cells on a well-stained cytospin preparation, or the presence of cranial nerve palsies at leukemia diagnosis. If blasts were identified in CSF cytospin preparations with a CSF cell count ≤ 5 cells/μL, the CNS status was classified as CNS2; in the case of traumatic lumbar puncture (TLP [≥10 red blood cells per microliter]) with the identification of blasts, the CNS status was categorized as TLP+, and as TLP− when no blasts were identified. Flow cytometric immunophenotyping was performed as previously described in the ALL-97 study^4^. Cytogenetic studies were performed using standard techniques. RT-PCR-based screening was conducted for major *BCR/ABL1*, minor *BCR/ABL1*, *ETV6/RUNX1*, *TCF3/PBX1*, *KMT2A/AFF1*, *KMT2A/MLLT4*, *KMT2A/MLLT3*, *KMT2A/MLLT1*, and *SIL/TAL1* fusion genes.

### Response and relapse criteria

Prednisolone responses were assessed after 7 days of monotherapy with prednisone and one intrathecal (IT) dose of methotrexate on day 1, and centrally reviewed in the study center. The presence of ≥1 × 10^9^ blasts/L in PB on day 8 was defined as a poor prednisolone response (PPR), while <1 × 10^9^ blasts/L was a prednisolone good response (PGR). BM responses were evaluated using aspiration smears on days 15 and 33 of the induction treatment. Complete remission (CR) was defined as <5% blasts in regenerating BM, the absence of leukemic blasts in PB and CSF, and no evidence of extramedullary disease. Resistance to therapy (non-response) was defined as not having achieved CR by the end of induction therapy (day 33). Relapse was defined as recurrence of ≥25% lymphoblasts in BM or localized leukemic infiltrates at any site.

### Risk stratification

Patients with non-T ALL were stratified into three risk groups according to the following criteria:

Extremely high risk (ER): B cell precursor (BCP)-ALL with PPR and/or evidence of t(4;11) (or *KMT2A/AFF1*), hypodiploidy (≤44), and/or acute mixed lineage leukemia/acute unclassified leukemia.

High risk (HR): No ER criteria and initial WBC ≥ 10 × 10^9^/L, and/or age at diagnosis ≥10 years, and/or extramedullary involvement, and/or the presence of *TCF3/PBX1* and/or t(1;19).

Standard risk (SR): No HR/ER criteria, initial WBC < 10 × 10^9^/L, age at diagnosis between 1 and 9 years.

A flow chart illustrating risk stratification is presented in Supplementary Fig. 1.

### Treatment

**Fig. 1:**
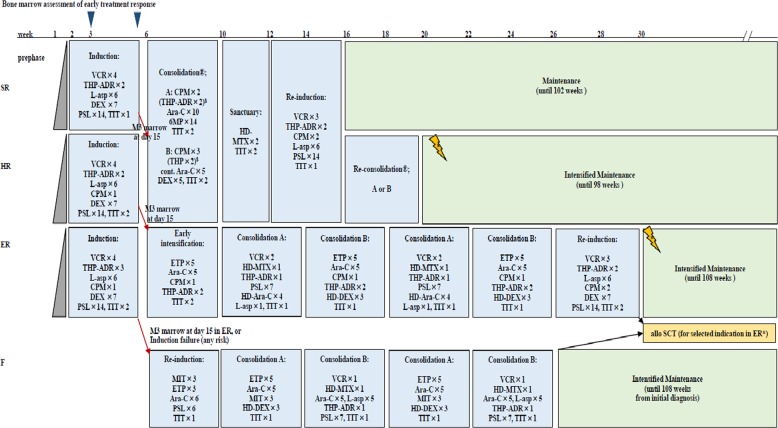
Outline of JACLS ALL-02 treatment. Details of treatment elements are listed in Table 1. The therapeutic irradiation dose for patients with initial central nervous system involvement was 12 Gy, irrespective of age. Prophylactic cranial radiotherapy was abolished for non-T cell ALL, irrespective of initial white blood cell count. SR standard risk, HR high risk, ER extremely high risk, PSL prednisone, VCR vincristine, DNR daunorubicin, THP pinorubin, ASP,*Escherichia coli* l-asparaginase, MTX methotrexate, 6-MP 6-mercaptopurine, ARA-C cytarabine, CPM cyclophosphamide, DEX dexamethasone, DOX doxorubicin, 6-MP 6-mercaptopurine, HD high dose, IT intrathecal, TIT triple intrathecal therapy, G-CSF granulocyte colony-stimulating factor, MT maintenance therapy, SCT stem cell transplantation, pCRT prophylactic cranial radiotherapy. Patients enrolled in JACLS ALL-02 were allocated to three risk groups using the modified NCI criteria, cytogenetics, and treatment responses. Bone marrow examinations were performed on days 15 and 33. Slow early responders, showing M3 marrow (blasts ≥ 25%) on day 15, were shifted to a higher risk group after induction therapy, as augmented post-induction therapy: SR to HR, HR to ER, and ER to F, respectively. Patients who did not achieve complete remission on day 33 were excluded from this study. Patients allocated to ER underwent allogeneic hematopoietic stem cell transplantation if they had an HLA-matched sibling donor.

The treatment strategy is shown in Fig. 1, and the details of treatment elements for each risk group are provided in Supplementary Tables 1–3. Patients showing M1 marrow (blasts <5%) on day 33 with M3 marrow (≥25%) in day 15 BM were assigned to a higher risk group after induction therapy and received augmented therapy as a post-induction treatment. Since excellent outcomes were observed in a previous study (OCLSG-94) using continuous cytarabine infusion in the consolidation phase for treatment of BCP-ALL^7^, a randomization trial was performed to test the superiority of a low-dose continuous cytarabine infusion over conventional repeated cytarabine injections (truncated BFM-typed Ib) as consolidation therapy in the SR and HR groups. Patients achieving CR at the end of induction (day 33) proceeded to randomization. Patients assigned to ER were candidates for allogeneic hematopoietic cell transplantation (HCT) by the end of the early phase, if HLA-matched siblings were available. Patients who failed to reach remission induction by day 33 received salvage chemotherapy (F protocol)^8^, followed by allogeneic HCT. The treatment protocol was amended with a reduction in the dose of pirarubicin from 25 to 20 mg/m^2^/dose during the induction phase because of a slight increase in regimen-related infections from JACLS ALL-97 since 18 June 2005. Treatment duration was predefined as 24 months in all risk groups, irrespective of sex.

In JACLS ALL-97, pCRT was performed for HR or ER patients, corresponding to NCI-HR, at a dose of 18 or 15 Gy for patients older or younger than 6 years of age, respectively^4^. By contrast, in JACLS ALL-02, pCRT was abolished irrespective of age and initial WBC in non T cell-ALL. Instead, protracted IT chemotherapy was introduced in JACLS ALL-02; triple IT (TIT) was administered eight times for SR/IR, 10 for HR, and 13 for ER in JACLS ALL-97, whereas TIT was administered 12 times for SR and 15 for HR/ER in JACLS ALL-02. Patients with CNS involvement received CRT at a dose of 18 Gy in JACLS ALL-97 and 12 Gy in JACLS ALL-02, irrespective of age. Intrathecal therapy for patients with CNS2 or TLP+ was the same as that for the patients with CNS1 in JACLS ALL-02.

### Historical control group

To evaluate the impact of the new JACLS ALL-02 risk stratification and treatment modification over those of the former JACLS ALL-97 trial^4^, the outcomes of patients enrolled in each study were compared, according to NCI risk criteria^9^. In historical comparisons of matched subgroups, patients in JACLS ALL-97 and ALL-02 were reclassified by the NCI risk criteria (“NCI-SR-97,” “NCI-HR-97”, “NCI-SR-02”, and “NCI-HR-02”). To evaluate the effects of omission of pCRT, JACLS ALL-97 patients in the risk groups “NCI-SR-97” and “NCI-HR-97” with B-ALL and without initial CNS involvement served as a historical control group.

### Statistical analysis

The primary endpoint for all comparisons was the 4yr-EFS rate: the time from the initiation of treatment to the earliest relapse, second malignancy, death from any cause, or last clinical contact. Failure included relapse, induction failure, secondary malignancies, and death. Four-year overall survival (OS), the secondary endpoint, was computed from the date of the start of induction therapy until the date of death or the last known date when the patient was alive (censored observation). Failure during induction was defined as non-CR at the end of induction therapy. In patients who failed to reach CR by the end of the induction phase, EFS was set to the first day. The Kaplan–Meier method was used to estimate the probability of EFS and OS, standard errors were obtained using the methods of Peto et al., and comparisons between groups were conducted using a two-sided log rank test. Confidence intervals were calculated according to Greenwood’s formula. A multivariate analysis of survival was performed using Cox’s proportional-hazard model to evaluate treatment effects with adjustments for stratification factors. All analyses were performed according to the intent-to-treat principle. Cumulative incidence (CI) for competing events were constructed using the method of Kalbfleisch and Prentice, and compared using the Gray test. Comparisons of randomized groups were performed as intent-to-treat. Differences in the distributions of individual parameters among patient subsets were analyzed using the *χ*^2^ test for categorical variables and the Mann–Whitney *U* test for continuous variables. A *p* value <0.05 was considered to indicate significance; all tests were two-tailed.

SR and HR patients were randomly assigned to either receive the truncated BFM-type Ib (arm A) regimen or low-dose cytarabine-containing regimen (arm B) at consolidation. According to the findings of previous studies, HR patients either received arm A or B as re-consolidation at the end of re-induction. The sample size was independently derived, based on the primary endpoint of EFS, in SR and HR. The probabilities of long-term EFS in SR and HR patients treated with the truncated BFM-typed Ib (arm A) regimen were estimated to be 85% and 70%, respectively. To detect an increase of 10%, 324 and 682 patients needed to be randomized in SR and HR, respectively (*α* = 0.05, *β* = 0.2).

Statistical analyses were conducted using the STATA program (version 11.0; StataCorp LP, College Station, TX). All patient data were updated in March 2015.

## Results

### Patient characteristics and risk assignment

Of the 1252 patients enrolled in JACLS ALL-02, seven were excluded from efficacy analyses: one protocol violation, three Ph+ ALL, two misdiagnoses, and one at the patient’s request. Patients with T cell ALL (*n* = 107) were excluded from the analysis because they were treated using an independent protocol. Among the remaining patients, 1138 were eligible for analysis in the present study.

**Fig. 2:**
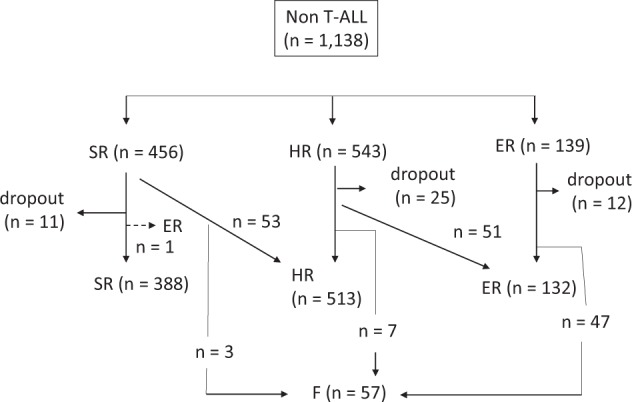
Consort diagram for 1138 non-T acute lymphoblastic leukemia (non-T ALL) patients treated in the Japan Association of Childhood Leukemia Study Group (JACLS) ALL-02 study. Non-T ALL patients (*n* = 1138) were stratified into standard risk (SR), high risk (HR), and extremely HR (ER) groups, based on age, initial white blood cell count, and initial response to prednisolone. Then, patients were re-stratified into SR, HR, ER, and failure (F) groups based on Day 15 and Day 33 bone marrow status.

Among the 1138 patients, 456 were provisionally stratified into SR, 543 to HR, and 139 to ER. After induction therapy, ten patients with t(4;11) and four with hypodiploidy were reassigned to receive the F protocol after the induction phase. Among these patients, 162 showing M3 marrow on day 15 were treated with regimens for higher risk disease than those initially assigned (53 SR to HR, 51 HR to ER, and 47 ER to F). Of 1138 patients, 56 (4.9%) received HCT at first CR; 3, 9, and 44 from the SR, HR, and ER groups, respectively. A Consort diagram of the risk stratification of the 1138 patients is presented in Fig. 2. Twenty-five of the 1138 patients exhibited extramedullary involvement: 23 with CNS and 2 with testicular involvement. Of these, no patient had both CNS and testicular involvement at diagnosis.

### Treatment outcome and survival analysis

CR was achieved in 96.4% of evaluable patients. Thirty-eight patients failed to achieve remission until the end of remission induction therapy; 2 of these 38 patients died of leukemia-associated complications in the induction phase. Among the 36 patients who were not in remission by protocol day 33, 17 were treated with the F protocol (Fig. 1) and 16 of these 17 (94%) patients attained CR using the F protocol.

Two patients developed secondary neoplasms (one acute myeloid leukemia and the other renal carcinoma) at a median time of 91.1 months (range, 54.3–127.8 months). Both of these neoplasms occurred after allogeneic HCT in first CR; these patients were still alive at last follow-up.

**Fig. 3:**
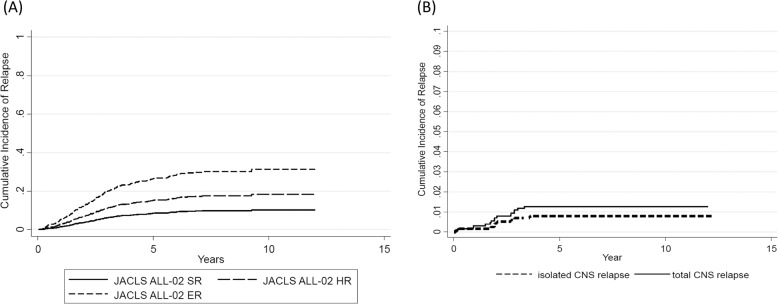
Cumulative incidence (CI) of CNS relapse. **a** CI of CNS relapse, according to allocated risk group. **b** CI of isolated CNS relapse (dashed line) and total CNS relapse (solid line).

For the 1138 evaluable patients, 4yr/8yr-EFS rate (±SE) was 85.4/82.0% (±1.0/1.2%) (95% CI: 83.3–87.4/79.6–84.3), while 4yr/8yr-OS was 91.2/88.6% (±0.8/1.0%) (95% CI: 89.6–92.9/86.7–90.6). In the intention-to-treat analysis, stratification by the JACLS ALL-02 risk criteria for evaluable non-T ALL resulted in distinct groups with 4yr/8yr-EFS of 90.3/88.4% (95% CI: 87.2–92.8/84.8–91.1) for SR (*N* = 456, 40.1%), 84.9/80.3% (81.6–87.7/76.4–83.6) for HR (*N* = 543, 47.7%), and 66.5/60.5% (57.9–73.7/51.5–68.5) for ER (*N* = 139, 12.2%) (log rank *p* < 0.0001) (Table 1). The CI at 4 years (4yr-CI) of relapse was 15.9% ( ± 1.0%) (95% CI: 14.0–18.0). CI rates for each risk group were as follows: SR, 8.7 ± 1.4% (95% CI: 6.3–12.1), HR, 13.4 ± 1.7% (95% CI: 10.5–17.1), and ER, 30.4 ± 5.3% (95% CI: 21.7–42.6) (Fig. 3a). BM was the most frequent site of relapse in all risk groups. Of 1138 patients, 16 relapsed with CNS involvement; 9 (0.8%) had isolated CNS relapse, and 7 (0.6%) had combined (BM + CNS) relapse. The 4yr-CI rates for isolated CNS relapse and for all relapses with CNS involvement were 0.9% (±0.3%) (95% CI: 0.44–1.6) and 1.5% (±0.4%) (95% CI: 0.92–2.4), respectively (Fig. 3b). The 4yr-CI of CNS relapse was higher in ER (isolated CNS relapse 2, combined CNS relapse 3; 4.3 ± 1.9%, 95% CI: 1.8–10.3) than in SR (isolated CNS relapse 3, combined CNS relapse 1; 0.9 ± 0.5%, 95% CI: 0.34–2.4) and HR (isolated CNS relapse 4, combined CNS relapse 3; 1.4 ± 0.52%, 95% CI: 0.65–2.9).

**Table 1:** Characteristics and outcomes of patients enrolled in the JACLS ALL-02 study (total *n* = 1138).

	*n*	4yr-EFS (%)	95% CI	Log rank *p*	4yr-OS (%)	95% CI	Log rank *p*
All eligible patients	1138	85.4	83.3–87.4		91.2	89.6–92.9	
NCI-SR	815	88.2	85.7–90.2	<0.0001	94.5	92.7–95.9	<0.0001
NCI-HR	323	76.4	71.3–80.6		83.1	78.5–86.8	
ALL-02 SR	456	90.4	87.2–92.8	<0.0001	97.3	95.3–98.5	<0.0001
ALL-02 HR	543	84.9	81.6–87.7		89.3	86.3–91.6	
ALL-02 ER	139	66	57.5–73.3		78.9	71.1–84.8	
Prednisolone response							
PGR	1041	87.1	85.0–89.1	<0.001	92.2	90.5–93.8	<0.001
PPR	97	66.2	56.6–75.8		81	73.1–88.9	
Treatment according to upgraded risk based on BM at day 15							
Upgraded	162	72	64.4–78.3	<0.0001	84.4	77.8–89.2	<0.0001
Not upgraded	928	89.8	87.8–91.6		94.6	92.9–95.9	
Sex							
Male	599	83.1	79.8–85.9	0.11	91	88.4–93.1	0.38
Female	539	86.7	83.5–89.3		91.5	88.8–93.6	
Age at diagnosis (years)							
1–9	923	87.5	85.1–89.5	<0.0001	94	92.2–95.3	<0.0001
10–18	215	73.4	67.0–78.8		79.8	73.7–84.6	
WBC at onset (/μL)							
<10,000	666	86.9	84.1–89.3	0.0005	93.6	91.4–95.2	0.0001
10,000–50,000	334	84.9	80.5–88.3		90	86.2–92.8	
50,000–100,000	72	80.5	69.3–88.0		87.5	77.4–93.3	
≥100,000	66	68	55.2–77.8		78.5	66.4–86.9	
Immunophenotype							
B cell precursor	1086	85.8	83.4–87.9	<0.0001	92	90.3–93.5	<0.0001
Mixed/Unclassified	52	63.5	48.9–74.9		74.7	60.4–84.5	
CNS status							
CNS1	949	86	83.8–88.2	0.54	91.7	89.9–93.4	0.4
CNS2	27	77.6	61.8–93.4		88.6	76.4–100	
CNS3	23	77.8	60.5–95.0		82.1	66.2–98.1	
Unknown	139	83	76.6–89.3		90.3	85.3–95.3	
TLP							
TLP+	6	33.3	0–71.1	<0.001	50	10.0–100	0.004
TLP−	42	87.7	77.6–97.8		90	80.7–99.3	
Cytogenetics/chimeric fusion							
Normal	368	83.8	80.0–87.6	<0.001	90.1	87.0–93.2	<0.001
Hyperdiploid	222	89.4	85.3–93.5		94.9	92.0–97.9	
*ETV6-RUNX1*	205	94.4	89.0–96.1		97.5	94.0–98.9	
Hypodiploid	4	25	0–67.4		50	1.0–99.0	
11q23 rearrangement/*KMT2A*-related fusion	22	81.3	64.8–97.9		85.6	70.6–100	
* TCF3-PBX1*/t(1;19)	85	84.7	77.1–92.4		87	79.8–94.2	
Other abnormalities	128	74.1	66.5–81.7		81	74.2–87.7	
Karyotypic failure	104	81.6	73.9–89.3		94.2	89.6–98.7	

*EFS* event-free survival, *CI* confidence interval, *OS* overall survival, *NCI* National Cancer Institute, *SR* standard risk, *HR* high risk, *ER* extremely high risk, *PGR* prednisolone good response, *PPR* prednisolone poor response, *BM* bone marrow, *WBC* white blood cell, *CNS* central nervous system, *TLP* traumatic lumbar puncture.

**Fig. 4:**
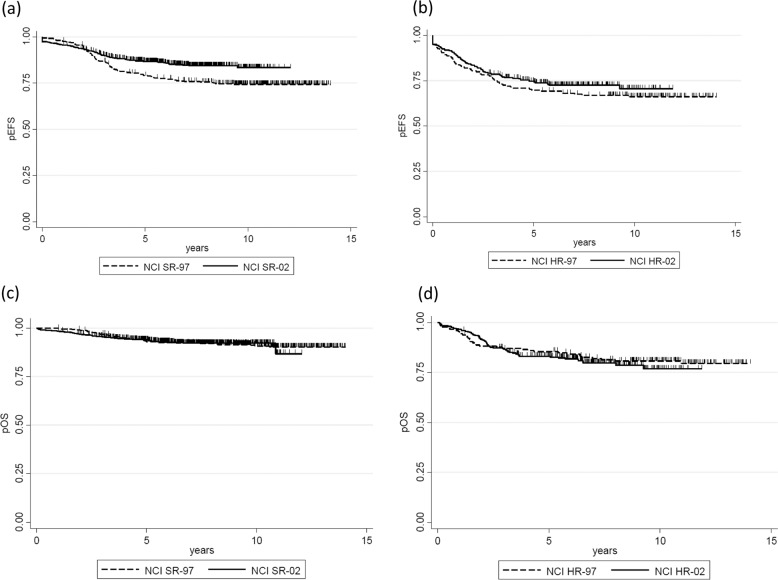
Kaplan–Meier estimate of evaluable patients reclassified according to NCI risk criteria. pEFS probability of event-free survival, pOS probability of overall survival. **a** pEFS in NCI-SR-97 (dashed line) vs. NCI-SR-02 (solid line). **b** pEFS in NCI-HR-97 (dashed line) vs. NCI-HR-02 (solid line). **c** pOS in NCI-SR-97 (dashed line) vs. NCI-SR-02 (solid line). **d** pOS in NCI-HR-97 (dashed line) vs. NCI-HR-02 (solid line).

To compare the treatment outcomes of JACLS ALL-02 to those of ALL-97, the outcomes of patients enrolled in each study were compared according to NCI risk criteria. Since Ph+ ALL was only included in ALL-97, it was excluded from JACLS ALL-97 for this analysis. Four-year EFS for NCI-SR-02 (*N* = 815) was 88.2% (±1.2%) (95% CI: 85.7–90.2), whereas that for NCI-SR-97 (*N* = 383) was 81.3% (±2.0%) (95% CI: 77.0–84.8) (log rank *p* = 0.0004) (Fig. 4a); however, 4yr-EFS for NCI-HR-02 (*N* = 323) was 76.4% (±2.4%) (95% CI: 71.3–80.6), whereas that for NCI-HR-97 (*N* = 179) was 71.0% (±3.4%) (95% CI: 63.7–77.0) (log rank *p* = 0.26) (Fig. 4b). Four-year OS for NCI-SR-02 (*N* = 814) was 94.5% (±1.1%) (95% CI: 92.2–96.8), whereas that for NCI-SR-97 (*N* = 383) was 95.0% (±0.8%) (95% CI: 92.7–95.9) (log rank *p* = 0.95) (Fig. 4c). Further, 4yr-OS for NCI-HR-02 (*N* = 323) was 83.1% (±2.1%) (95% CI: 78.5–86.8), whereas that for NCI-HR-97 (*N* = 179) was 86.6% (±2.6%) (95% CI: 80.6–90.7) (log rank *p* = 0.52) (Fig. 4d). These findings suggest that pEFS of the NCI-SR group was improved in the ALL-02 trial; however, treatment outcomes for the NCI-HR group were not improved. Multivariate analysis revealed that the JACLS ALL-02 risk group, favorable cytogenetic/genetic abnormalities (*ETV6-RUNX1* + HHD), and unfavorable cytogenetic/genetic abnormalities (hypodiploid and abnormalities other than *ETV6-RUNX1*, HHD, *TCF3-PBX1*, normal karyotype, and 11q23/*KMT2A*-related fusion) were associated with pEFS (Table 2).

**Table 2:** Multivariate analysis to identify factors predicting inferior outcomes.

	EFS				OS			
	HR	95% CI		*p*	HR	95% CI		*p*
NCI-HR vs. SR	1.37	1	1.89	0.05	1.53	1.03	2.27	0.03
JACLS risk	1.46	1.1	2	0.02	1.88	1.28	2.77	0.001
*ETV6-RUNX1*+HHD (absent vs. present)	1.53	1.07	2.2	0.02	1.79	1.11	2.91	0.02
Hypodiploid+other abnormalities^a^ (absent vs. present)	0.6	0.44	0.92	0.01	0.59	0.38	0.91	0.02
PPR vs. PGR	1.38	0.84	2.27	0.21	0.93	0.51	1.7	0.82

*EFS* event-free survival, *OS* overall survival, *HR* hazard ratio, *CI* confidence interval, *NCI* National Cancer Institute, *HR* high risk, *SR* standard risk, *PGR* prednisolone good response, *PPR* prednisolone poor response.

^a^Cytogenetic/genetic abnormalities other than ETV6-RUNX1, TCF3-PBX1, high hyperdiploid, 11q23 rearrangement/KMT2A-related fusion, normal karyotype, and karyotypic failure.

CNS status at diagnosis data were available for 999 of 1138 patients, who were categorized as CNS1 (*N* = 949, 83%), CNS2 (*N* = 27, 2.4%), and CNS3 (*N* = 23, 2%). In addition, 48 of these 999 patients (4.8%) had TLP at initial diagnosis. Further, 6 of 48 patients (12.5%) were TLP+. CNS status of 139 patients (12.2% of 1138 patients) was not determined because the detailed data could not be collected from each institute. Although there was no significant difference in 4-year EFS and OS according to CNS status, 4yr-EFS in patients with TLP+ at diagnosis (*n* = 6) was 33.3% (95% CI: 0–71.1), while that with TLP− at diagnosis (*n* = 42) was 87.7% (95% CI: 77.6–97.8) (log rank *p* < 0.001) (Table 1).

### Toxicities

Fourteen (1.2%) patients died during the 5-week induction: three SR, three HR, and one ER patient died of sepsis; three HR and one ER patient of fungal infection; and one HR patient of acute pancreatitis due to l-asparaginase; one ER patient of cerebral hemorrhage; and one ER patient of hepatic failure. Seven patients (0.6%) died after achievement of CR because of treatment complications; the mortality rate was the highest in ER, with four (2.9%) deaths, all resulting from allogenic HCT-related complications. Three patients who died in CR were treated as SR and HR; one died in the induction phase and two during maintenance due to infection or treatment-related complications.

Among 388 definite SR patients, five suffered from grade 4 infection in induction therapy and two in subsequent therapies. Among 513 definite HR patients, six suffered from grade 4 infection in induction therapy and two in subsequent therapies. Among 130 definite ER patients, three suffered from grade 4 infection in induction therapy.

Hepatic toxicity was the most common grade 4 complication; incidence rates among SR, HR, and ER patients were 22.9%, 13.1%, and 5.3%, respectively. Pancreatitis (grades 3 and 4) was observed in 2 of 388 definite SR patients, 7 of 513 definite HR patients, and 3 of 130 definite ER patients. Allergy (grade 4) was detected in 1 of 388 SR patients, 7 of 513 definite HR patients, and 1 of 132 definite ER patients. l-ASP intolerance was observed in 4.4%, 9.0%, and 10% of SR, HR, and ER patients, respectively. Major causes of l-asp intolerance were allergic reaction and pancreatitis.

### Randomized study to assess the impact of low-dose continuous cytarabine infusion as consolidation therapy

Among 388 patients stratified into definite SR, 200 and 188 patients were randomized to the A and B arms, respectively. Among 513 patients stratified into definite HR, 251 and 257 patients were randomized to arms A and B, respectively. Data on randomization were not available for 5 of the 513 HR patients; hence these patients were excluded from this analysis. The two groups had similar pretreatment characteristics (Supplementary Table 4). The treatment outcomes of the randomized trial were assessed with a median follow-up of 6.6 years. In the definite SR group, 4yr-EFS was 89.7% (95% CI: 84.5–93.2) in arm A and 93.4% (95% CI: 88.7–96.2) in arm B; there was no significant difference between the two arms (log rank *p* = 0.7). OS rates at 5 and 10 years also did not differ significantly (98.0% [94.7–99.2%] vs. 97.2% [93.3–98.8%] at 5 years and 95.0% [90.1–97.5%] vs. 95.4% [90.4–97.8%] at 10 years, log rank *p* = 0.85). In the definite HR group, 4yr-EFS rates were 89.9% (95% CI: 85.4–93.0) in arm A and 90.5% (95% CI: 86.1–93.4) in arm B, which was not a significant difference (log rank *p* = 0.97). OS rates at 5 and 10 years were also not significantly different (93.5% [89.5–95.9%] vs. 92.8% [88.8–95.4%] at 5 years and 95.0% [90.1–97.5%] vs. 89.2% [80.6–94.2%] at 10 years; log rank *p* = 0.79).

## Discussion

JACLS ALL-02 included a large unselected population of 1138 evaluable patients with non-T ALL. The 4yr-OS rate was 91.2%, which is comparable to that of these major studies reported to date (Table 3)^10–14^. In particular, 388 of 1138 (34.1%) patients were treated with the less intensive ALL-02 SR protocol, which comprised only two cycles of HD-MTX (3 g/m^2^) and one course of re-induction therapy (VCR, l-asp, pirarubicin, and prednisolone). In terms of NCI-SR patients (*n* = 815), 388 (47.6%) were treated with the ALL-02 SR protocol. In addition, the profile of toxicities, such as the incidence of induction death, was comparable to that in another major clinical trial^15^. The present study, which includes the largest childhood ALL cohort ever reported in Japan, documents that EFS in the NCI-SR group was significantly better than that in the former JACLS ALL-97 trial. We attribute the superior outcomes to the incorporation of proper intensification of the ALL-97-SR and IR protocols, particularly the early intensification, re-induction, and re-consolidation phase, coupled with stringent risk classification, based on prednisolone responses and early BM responses on day 15 in the induction phase.

**Table 3:** Comparison of treatment outcomes for pediatric non-T ALL treated with a contemporary protocol.

	*n*	% EFS (years)	% OS (years)
JACLS ALL-02	1138	84.8 (4)	91.2 (4)
NCI-SR	815	88.2 (4)	94.5 (4)
NCI-HR	323	76.4 (4)	83.1 (4)
1–9 years	923	87.5 (4)	94 (4)
10–18 years	215	73.4 (4)	79.8 (4)
JACLS ALL-97	566	77 (10)	87 (10)
NCI-SR	383	81.3 (4)	95 (4)
NCI-HR	179	71.0 (4)	86.6 (4)
BFM2000	4016	80.4 (7)	91.8 (7)
BFM95	1798	80.2 (6)	
NCI-SR	1256	86.5 (6)	
NCI-HR	512	67.4 (6)	
St. Jude Total XV			
NCI-SR	258	89.7 (5)	97.7 (5)
NCI-HR	164	82.4 (5)	89.7 (5)
NOPHO ALL2008			
1–9 years	1022	89 (5)	94 (5)
10–17 years	266	80 (5)	87 (5)
TCCSG L04-16		80.1 (5)	91.9 (5)
NCI-SR	646	82.9 (5)	94.2 (5)
NCI-HR	265	73.2 (5)	86.2 (5)

*EFS* event-free survival, *OS* overall survival, *JACLS* Japan Association of Childhood Leukemia Study Group, *NCI* National Cancer Institute, *SR* standard risk, *HR* high risk, *BFM* Berlin–Frankfurt–Munster, *NOPHO* Nordic Society for Pediatric Hematology and Oncology, *TCCSG* Tokyo Children’s Cancer Study Group.

Conversely, EFS of NCI-HR patients was not improved in the JACLS ALL-02 trial, demonstrating that treatment intensification in the ALL-02 study, including intensified early consolidation, re-induction, and a re-consolidation phase using more cyclophosphamide and cytarabine in the HR protocol, and intensified induction, early intensification phase using more anthracycline agent, and high-dose DEX, in addition to HCT in CR1, in the ER protocol, was insufficient for NCI-HR patients.

We conducted a randomized controlled trial comparing the efficacy of low-dose continuous cytarabine infusion with conventional cytarabine block in the consolidation phase in the SR/HR groups because of the excellent outcome reported for continuous cytarabine infusion in the OCLSG-94 trial^7^; however, there was no significant difference in pEFS or pOS between the two arms. Recent studies have revealed that subtypes of B-ALL associated with poor prognosis, such as Ph-like ALL^16–18^, *IKZF1*-plus ALL^19^, and *MEF2D*-rearranged ALL^20,21^, comprise a substantial proportion of B-ALL in the NCI-HR category. Consistent with these findings, we previously reported poor prognosis for patients with B-ALL with *IKZF1* deletion in the JACLS ALL-02 study, particularly in NCI-HR or PPR patients^22,23^. Thus, new therapeutic options, such as molecular targeting therapy and immunotherapy, should be implemented to improve the outcome of patients in the NCI-HR group^24–26^.

Although our risk stratification system appears to function well, a limitation of this study is that the JACLS ALL-02 protocol was not MRD-driven. Recent studies have demonstrated that precise risk stratification, based on MRD, improves outcomes of pediatric ALL^11,27^, even for patients with poor prognostic subtypes, such as Ph-like ALL^28^. Thus, it is possible that proper intensification of treatment of MRD-positive patients could improve the outcome of patients stratified into definite SR or HR with positive MRD. Therefore, the next national protocol for pediatric B-ALL is planned to include a PCR-MRD-based risk stratification system.

In addition, karyotypic analysis of 104 patients (9.1%) was unsuccessful. Although the RT-PCR-based screening of chimeric fusion genes could identify genetic subgroups in some of 104 patients, DNA index by flow cytometric analysis might be mandatory for the determination of genetic subgroups, especially high hyperdiploid or hypodiploid ALL.

A recent systematic review and meta-analysis of 47 randomized trials of CNS-directed therapy, conducted between the 1970s and 1990s, showed that CRT may generally be replaced by IT therapy^29^. Moreover, meta-analyses of individual participant data by major collaborative ALL study groups have also concluded that CRT does not impact the risk of relapse in children^30^. Although CNS status of 139 patients (12.2%) was not determined, the 4yr-CI of CNS relapse of 1.5% for non-T ALL in JACLS ALL-02 was a welcome finding; however, CRT was restricted to patients with CNS3 at diagnosis. Together with the findings of previous meta-analyses, the results of the present study reinforce the view that, with the intensification of systemic and IT chemotherapies, pCRT may be omitted without compromising OS in non-T ALL. Although CRT was associated with a reduced risk of isolated or combined CNS relapse in patients with CNS3 at diagnosis in our trial, CRT inevitably increases a wide range of complications, including neuropsychological sequelae, endocrinopathy, and second cancers^29^. Thus, CRT may not be warranted, even in the poor-risk subgroup. Although remarkably low isolated (0.9%) and total (1.5%) CNS relapse rates were achieved in the present study, the complete abolition of pCRT led to the identification of risk factors for CNS relapse in non-T ALL. Gajjar et al.^31^ reported that CNS2 at diagnosis was one risk factor, in addition to TLP with leukemic blasts at diagnosis, in the Total Therapy series cohort at St Jude Children’s Research Hospital. We also found that patients identified as TLP+ at diagnosis showed inferior EFS in our study. Jastaniah et al.^32^ reported that the prognosis of TLP+ pediatric ALL was improved when patients were administered intensified IT therapy in the induction phase. Thus, patients with TLP+ receive augmented IT therapy in the ongoing national protocol, JPLSG B12 (ref. ^33^), in Japan.

In summary, refined stratification and risk-adjusted therapy in JACLS ALL-02 resulted in improved EFS of NCI-SR patients. New therapeutic strategies, including molecular targeting agents, immunotherapy, and innovative cell therapy, are needed to improve the outcome of NCI-HR patients.

## Supplementary information

supplementary Table 1-4

suplementary Figure
