# Supplementary material for: Risk-adjusted therapy for pediatric non-T cell ALL improves outcomes for standard risk patients: results of JACLS ALL-02
Source: Blood Cancer J. 2020 Feb 27;10(2):23. doi: 10.1038/s41408-020-0287-4 (PMC7046744; doi:10.1038/s41408-020-0287-4)
Supplement: Supplementary file 1 — supplementary Table 1-4 [file 41408_2020_287_MOESM1_ESM.docx]

Supplemental Table 1. JACLS ALL-02 treatment protocol SR patients

| Treatment element/drug | Single or daily dose | Days of application per element^a^ |
| --- | --- | --- |
| Pre-phase |  |  |
| Prednisolone | 60 mg/m^2^/d | 1–7 |
| Methotrexate | 12 mg/dose^b^ | 1 |
|  |  |  |
| Induction |  |  |
| Vincristine | 1.5 mg/m^2^ iv | 8, 15, 22, 29 |
| Pirarubicin | 20 mg/m^2^ div over 1 h | 8, 9 |
| L-asparaginase | 6,000 IU/m^2^ div over 4 h or im | 15, 17, 19, 22, 24, 26 |
| Dexamethasone | 10 mg/m^2^ div over 1 h | 8–14 |
| Prednisolone | 40 mg/m^2^ po or div over 1 h | 15–28 and taper^d^ |
| TIT^c^ |  | 8^e^ |
|  |  |  |
| Consolidation  (randomization) |  |  |
| Arm A |  |  |
| Cyclophosphamide | 750 mg/m^2^ div over 1 h | 36, 43 |
| Cytarabine | 75 mg/m^2^ div over 1 h | 36–41, 43–48 |
| 6-mercaptopurine | 50 mg/m^2^ po | 36–49 |
| TIT |  | 36, 43 |
|  |  |  |
| Arm B |  |  |
| Cyclophosphamide | 500 mg/m^2^ div over 1 h | 36, 38, 40 |
| Cytarabine | 100 mg/m^2^ cont iv | 36–40 |
| Dexamethasone | 10 mg/m^2^ div over 1 h | 36–40 |
| TIT |  | 36, 43 |
|  |  |  |
| Sanctuary |  |  |
| Methotrexate | 3000 mg/m^2^/dose div over 24 h^f^ | 64, 71 |
| Leucovorin | 15 mg/m^2^ iv x 6 q6 h | Start at 36 h from the beginning of methotrexate |
| TIT |  | 58, 65 |
|  |  |  |
| Re-induction |  |  |
| Vincristine | 1.5 mg/m^2^ iv | 71, 78, 85 |
| Pirarubicin | 25 mg/m^2^ div over 1 h | 71, 78 |
| L-asparaginase | 6,000 IU/m^2^ im | 71, 73, 75, 78, 80, 82 |
| Prednisolone | 40 mg/m^2^ po or div over 1 h | 71–84 and taper^g^ |
| TIT^c^ |  | 71^e^ |
|  |  |  |
| Maintenance |  |  |
| 6-mercaptopurine | 50 mg/m^2^ po | 1–28 |
| Methotrexate | 25 mg/m^2^ po | 1, 8, 15, 22 |
| Vincristine | 1.5 mg/m^2^ iv | 22 |
| Prednisolone | 40 mg/m^2^ po | 22–28 |
| Repeat this therapy 20 times |  |  |

^a^Time schedule adjustments were allowed if clinical condition and bone marrow recovery were inadequate.

^b^Doses of IT drugs were adjusted for children < 3 years of age.

^c^TIT: intrathecal therapy with methotrexate, cytarabine, and hydrocortisone.

^d^Steroid doses were tapered over 7 additional days.

^e^Additional IT therapy on days 10 and 15 (BFM) was administered to patients with CNS3 status.

^f^A loading dose of 10% was infused over 30 min, with the remaining 90% over 23.5 h. Leucovorin rescue was given at 42, 48, and 54 h (15 mg/m^2^ each). Doses of leucovorin rescue were adjusted if MTX levels were > 1.0 µmol/l at 42 h or later. If the MTX level at 54 h was > 0.1 µmol/l, rescue was continued at 6 hour intervals until MTX levels were ≤ 0.1 µmol/l.

^g^Steroid doses were tapered over 3 additional days.

Abbreviations: SR, standard risk; iv, intravenous infusion; im, intramuscular infusion; div, intravenous infusion by drip; po, per oral; cont iv, continuous intravenous infusion.

Supplemental Table 2. JACLS ALL-02 treatment protocol for HR patients

| Treatment element/drug | Single or daily dose | Days of application per element^a^ |
| --- | --- | --- |
| Pre-phase |  |  |
| Prednisolone | 60 mg/m^2^/d | 1–7 |
| Methotrexate | 12 mg/dose^b^ | 1 |
|  |  |  |
| Induction |  |  |
| Vincristine | 1.5 mg/m^2^ iv | 8, 15, 22, 29 |
| Pirarubicin | 20 mg/m^2^ div over 1 h | 8, 9 |
| L-asparaginase | 6,000 IU/m^2^ div over 4 h or im | 15, 17, 19, 22, 24, 26 |
| Cyclophosphamide | 1,200 mg/m^2^ div over 1 h | 10 |
| Dexamethasone | 10 mg/m^2^ div over 1 h | 8–14 |
| Prednisolone | 40 mg/m^2^ po or div over 1 h | 15–28 and taper^d^ |
| TIT^c^ |  | 8^e^ |
|  |  |  |
| Consolidation  (randomization ) |  |  |
| Arm A |  |  |
| Cyclophosphamide | 750 mg/m^2^ div over 1 h | 36, 43 |
| Pirarubicin | 25 mg/m^2^ div over 1 h | 36, 37 |
| Cytarabine | 75 mg/m^2^ div 1 h | 36–41, 43–48 |
| 6-mercaptopurine | 50 mg/m^2^ po | 36–49 |
| TIT |  | 36, 43 |
|  |  |  |
| Arm B |  |  |
| Cyclophosphamide | 500 mg/m^2^ div over 1 h | 36, 38, 40 |
| Pirarubicin | 25 mg/m^2^ div over 1 h | 36, 37 |
| Cytarabine | 100 mg/m^2^ cont iv | 36–40 |
| Dexamethasone | 10 mg/m^2^ div over 1 h | 36–40 |
| TIT |  | 36, 43 |
|  |  |  |
| Sanctuary |  |  |
| Methotrexate | 3,000 mg/m^2^/dose div over 24 h^f^ | 64, 71 |
| Leucovorin | 15 mg/m^2^ iv x 6 q6 h | Start at 36 h from the beginning of methotrexate |
| TIT |  | 58, 65 |
|  |  |  |
| Re-induction |  |  |
| Vincristine | 1.5 mg/m^2^ iv | 78, 85, 92 |
| Pirarubicin | 25 mg/m^2^ div over 1 h | 78, 79 |
| L-asparaginase | 6,000 IU/m^2^ im | 78, 80, 82, 85, 87, 89 |
| Cyclophosphamide | 500 mg/m^2^ div over 1 h | 78, 85 |
| Prednisolone | 40 mg/m^2^ po or div over 1 h | 78–91 and taper^g^ |
| TIT^c^ |  | 78^e^ |
|  |  |  |
| Re-Consolidation  (randomization) |  |  |
| Arm A |  |  |
| Cyclophosphamide | 750 mg/m^2^ div over 1 h | 106, 113 |
| Pirarubicin | 25 mg/m^2^ div over 1 h | 71, 78 |
| Cytarabine | 75 mg/m^2^ div 1 h | 106–110, 113–117 |
| 6-mercaptopurine | 50 mg/m^2^ po | 106–119 |
| TIT |  | 106, 113 |
|  |  |  |
| Arm B |  |  |
| Cyclophosphamide | 500 mg/m^2^ div over 1 h | 106, 108, 110 |
| Pirarubicin | 25 mg/m^2^ div over 1 h | 106, 107 |
| Cytarabine | 100 mg/m^2^ cont iv | 106–110 |
| Dexamethasone | 10 mg/m^2^ div over 1 h | 106–110 |
| TIT |  | 106, 113 |
|  |  |  |
| Maintenance |  |  |
| 6-mercaptopurine | 50 mg/m^2^ po | 1–28, 57–77 |
| Methotrexate | 150 mg/m^2^ po | 1, 15, 29, 71, 85, 99 |
| Vincristine | 1.5 mg/m^2^ iv | 29, 36, 42, 85, 92, 98 |
| Cyclophosphamide | 600 mg/m^2^ div over 1 h | 36 |
| L-asparaginase | 10,000 IU/m^2^ im | 29, 36, 43, 85, 92, 99 |
| Pirarubicin | 25 mg/m^2^ div over 1 h | 92 |
| Prednisolone | 40 mg/m^2^ po | 29–42, 85–99 |
| Repeat this therapy 4 times |  |  |

^a^Time schedule adjustments were allowed if clinical condition and bone marrow recovery were inadequate.

^b^Doses of IT drugs were adjusted for children < 3 years of age.

^c^TIT: intrathecal therapy with methotrexate, cytarabine, and hydrocortisone.

^d^Steroid doses were tapered over 7 additional days.

^e^Additional IT therapy on day 10 and 15 (BFM) was administered to patients with CNS3 status.

^f^A loading dose of 10% was infused over 30 min, with the remaining 90% over 23.5 h. Leucovorin rescue was given at 42, 48, and 54 h (each 15 mg/m^2^). Doses of leucovorin rescue were adjusted, if MTX levels were > 1.0 µmol/l at 42 h or later. If the MTX level at 54 h was > 0.1 µmol/l, rescue was continued at 6 hour intervals until MTX levels were ≤ 0.1 µmol/l.

^g^Steroid doses were tapered over 3 additional days.

Abbreviations: HR, high risk; iv, intravenous infusion; div, intravenous infusion by drip; im, intramuscular infusion; po, per oral; cont iv, continuous intravenous infusion.

Supplemental Table 3. JACLS ALL-02 treatment protocol for ER patients

| Treatment element/drug | Single or daily dose | Days of application per element^a^ |
| --- | --- | --- |
| Pre-phase |  |  |
| Prednisolone | 60 mg/m^2^/d | 1–7 |
| Methotrexate | 12 mg/dose^b^ | 1 |
|  |  |  |
| Induction |  |  |
| Vincristine | 1.5 mg/m^2^ iv | 8, 15, 22, 29 |
| Pirarubicin | 20 mg/m^2^ div over 1 h | 8, 9, 10 |
| L-asparaginase | 6,000 IU/m^2^ div over 4 h or im | 15, 17, 19, 22, 24, 26 |
| Cyclophosphamide | 1200 mg/m^2^ div over 1 h | 10 |
| Dexamethasone | 10 mg/m^2^ div over 1 h | 8–14 |
| Prednisolone | 40 mg/m^2^ po or div over 1 h | 15–28 and taper^d^ |
| TIT^c^ |  | 8^e^ |
|  |  |  |
| Early intensification |  |  |
| Etoposide | 100 mg/m^2^ div over 2 h | 36–40 |
| Cytarabine | 300 mg/m^2^ div 4 h | 36–40 |
| Cyclophosphamide | 1200 mg/m^2^ div over 1 h | 38 |
| Pirarubicin | 25 mg/m^2^ div over 1 h | 37, 39 |
| TIT^c^ |  | 36, 43^e^ |
|  |  |  |
| Consolidation A1 |  |  |
| Vincristine | 1.5 mg/m^2^ iv | 64, 71 |
| Methotrexate | 3,000 mg/m^2^ div over 24 h | 64 |
| Leucovorin | 15 mg/m^2^ iv x 6 q6 h | Start at 36 h from the beginning of methotrexate |
| Pirarubicin | 25 mg/m^2^ div over 1 | 67 |
| Prednisolone | 40 mg/m^2^ po | 64–70 |
| Cytarabine | 3,000 mg/m^2^ div 3 h x 4 q12 h | 67–69 |
| L-asparaginase | 20,000 IU/m^2^ im | 69 |
| TIT^c^ |  | 65^e^ |
|  |  |  |
| Consolidation B1 |  |  |
| Etoposide | 100 mg/m^2^ div over 2 h | 92–96 |
| Cytarabine | 300 mg/m^2^ div 4 h | 92–96 |
| Cyclophosphamide | 1200 mg/m^2^ div over 1 h | 94 |
| Pirarubicin | 25 mg/m^2^ div over 1 h | 93, 95 |
| Dexamethasone | 100 mg/m^2^ div over 1 h | 92, 94, 96 |
| TIT^c^ |  | 92^e^ |
|  |  |  |
| Consolidation A2 |  |  |
| Vincristine | 1.5 mg/m^2^ iv | 120, 127 |
| Methotrexate | 3,000 mg/m^2^ div over 24 h | 120 |
| Leucovorin | 15 mg/m^2^ iv x 6 q6 h | Start at 36 h from the beginning of methotrexate |
| Pirarubicin | 25 mg/m^2^ div over 1 h | 123 |
| Prednisolone | 40 mg/m^2^ po | 120–126 |
| Cytarabine | 3,000 mg/m2 div 3 h × 4 q12 h | 123–125 |
| L-asparaginase | 20,000 IU/m^2^ im | 125 |
| TIT^c^ |  | 121^e^ |
|  |  |  |
| Consolidation B2 |  |  |
| Etoposide | 100 mg/m^2^ div over 2 h | 148–152 |
| Cytarabine | 300 mg/m^2^ div 4 h | 148–152 |
| Cyclophosphamide | 1,200 mg/m^2^ div over 1 h | 150 |
| Pirarubicin | 25 mg/m^2^ div over 1 h | 149, 151 |
| Dexamethasone | 100 mg/m^2^ div over 1 h | 148, 150, 152 |
| TIT^c^ |  | 148^e^ |
|  |  |  |
| Re-induction |  |  |
| Vincristine | 1.5 mg/m^2^ iv | 176, 183, 190 |
| Pirarubicin | 25 mg/m^2^ div over 1 h | 176, 183 |
| L-asparaginase | 6,000 IU/m^2^ im | 176, 178, 180, 183, 185, 187 |
| Cyclophosphamide | 500 mg/m^2^ div over 1 h | 176, 183 |
| Prednisolone | 40 mg/m^2^ po or div over 1 h | 176–189 and taper^g^ |
| TIT^c^ |  | 176, 183^e^ |
|  |  |  |
| Maintenance |  |  |
| 6-mercaptopurine | 50 mg/m^2^ po | 1–28, 57–77 |
| Methotrexate | 150 mg/m^2^ po | 1, 15, 29, 71, 85, 99 |
| Vincristine | 1.5 mg/m^2^ iv | 29, 36, 42, 85, 92, 98 |
| Cyclophosphamide | 600 mg/m^2^ div over 1 h | 36 |
| L-asparaginase | 10,000 IU/m^2^ im | 29, 36, 43, 85, 92, 99 |
| Pirarubicin | 25 mg/m^2^ div over 1 h | 92 |
| Prednisolone | 40 mg/m^2^ po | 29–42, 85–99 |
| Repeat this therapy 4 times |  |  |

^a^Time schedule adjustments were allowed if clinical condition and bone marrow recovery were inadequate.

^b^Doses of IT drugs were adjusted for children < 3 years of age.

^c^TIT: intrathecal therapy with methotrexate, cytarabine, and hydrocortisone.

^d^Steroid doses were tapered over 7 additional days.

^e^Additional IT therapy was administered to patients with CNS3 status on day 10 and 15 (BFM).

Abbreviations: ER, extremely high risk; iv, intravenous infusion; div, intravenous infusion by drip; im, intramuscular infusion; po, per oral; cont iv, continuous intravenous infusion.

Supplementary Table 4. Comparison of characteristics of patients in each randomization arm

| Definitive risk | SR | | | HR | | |
| --- | --- | --- | --- | --- | --- | --- |
| Arm | A | B | p | A | B | p |
| Sex |  |  |  |  |  |  |
| Male | 103 | 89 | 0.41 | 133 | 143 | 0.55 |
| Female | 97 | 99 |  | 118 | 11 |  |
| Age (years) at diagnosis |  |  |  |  |  |  |
| 1–4 | 153 | 147 | 0.7 | 137 | 136 | 0.68 |
| 5–9 | 47 | 41 |  | 45 | 54 |  |
| 10–18 | 0 | 0 |  | 69 | 67 |  |
| WBC at diagnosis (10^3^/μL) |  |  |  |  |  |  |
| < 10 | 200 | 187 | 0.3 | 143 | 130 | 0.25 |
| 10–49.9 | 0 | 1 |  | 92 | 99 |  |
| 50–99.9 | 0 | 0 |  | 10 | 18 |  |
| ≥ 100 | 0 | 0 |  | 6 | 10 |  |
| Fusion gene |  |  |  |  |  |  |
| *TCF3-PBX1* | 0 | 0 | 0.2 | 28 | 39 | 0.26 |
| *KMT2A-MLLT3* | 0 | 0 |  | 0 | 2 |  |
| *KMT2A-MLLT1* | 0 | 0 |  | 5 | 2 |  |
| *ETV6-RUNX1* | 40 | 49 |  | 46 | 41 |  |
| Chromosome number |  |  |  |  |  |  |
| ≤ 44 | 18 | 23 | 0.37 | 18 | 24 | 0.84 |
| 45 | 3 | 7 |  | 18 | 18 |  |
| 46 | 123 | 101 |  | 152 | 146 |  |
| 47–50 | 7 | 9 |  | 18 | 17 |  |
| ≥ 50 | 49 | 48 |  | 45 | 52 |  |
| Discontinuation of L-asp |  |  |  |  |  |  |
| yes | 8 | 9 | 0.71 | 27 | 23 | 0.49 |
| no | 192 | 179 |  | 224 | 234 |  |

SR, standard risk; HR, high risk; WBC, white blood cell count; L-asp, L-asparaginase.
