# Supplementary material for: Risk-adjusted therapy for pediatric non-T cell ALL improves outcomes for standard risk patients: results of JACLS ALL-02
Source: Blood Cancer J. 2020 Feb 27;10(2):23. doi: 10.1038/s41408-020-0287-4 (PMC7046744; doi:10.1038/s41408-020-0287-4)
Supplement: Supplementary file 2 — suplementary Figure [file 41408_2020_287_MOESM2_ESM.pptx]

## Slide 1
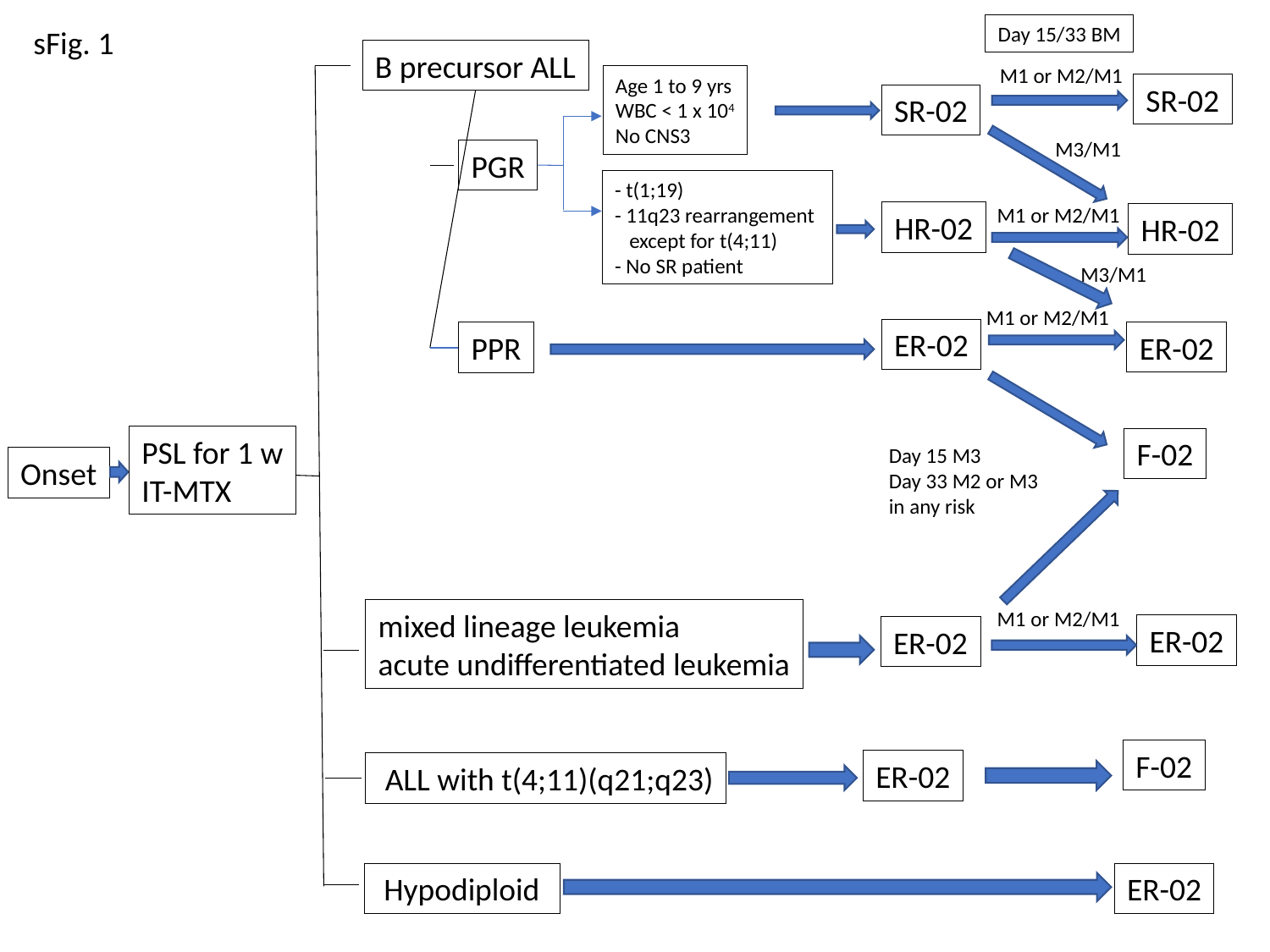

Day 15/33 BM
sFig. 1
B precursor ALL
M1 or M2/M1
Age 1 to 9 yrs
WBC < 1 x 10^4^
No CNS3
SR-02
PGR
- t(1;19)
- 11q23 rearrangement
 except for t(4;11)
- No SR patient
HR-02
ER-02
PPR
SR-02
M3/M1
M1 or M2/M1
HR-02
M3/M1
M1 or M2/M1
ER-02
PSL for 1 w
IT-MTX
Onset
F-02
Day 15 M3
Day 33 M2 or M3
in any risk
M1 or M2/M1
mixed lineage leukemia
acute undifferentiated leukemia
ER-02
ER-02
F-02
ER-02
 ALL with t(4;11)(q21;q23)
 Hypodiploid
ER-02
